# Complete Genome Sequences of *Enterococcus rotai* LMG 26678^T^ and *Enterococcus silesiacus* LMG 23085^T^

**DOI:** 10.1128/genomeA.01387-16

**Published:** 2016-12-15

**Authors:** Ana C. Lauer, Ben W. Humrighouse, Vladimir Loparev, Patricia L. Shewmaker, Anne M. Whitney, John R. McQuiston, Richard W. McLaughlin

**Affiliations:** a Special Bacteriology Reference Laboratory, Bacterial Special Pathogens Branch, Division of High-Consequence Pathogens and Pathology, Centers for Disease Control and Prevention, Atlanta, Georgia, USA; b Division of Scientific Resources, Centers for Disease Control and Prevention, Atlanta, Georgia, USA; c Respiratory Diseases Branch, Division of Bacterial Diseases, Centers for Disease Control and Prevention, Atlanta, Georgia, USA; d General Studies, Gateway Technical College, Kenosha, Wisconsin, USA

## Abstract

The inclusion of molecular methods in the characterization of the novel species *Enterococcus horridus* necessitated the sequencing and assembly of the genomes of the closely related *Enterococcus rotai* and *Enterococcus silesiacus*. Sequencing using Illumina technology in combination with optical mapping led to the generation of closed genomes for both isolates.

## GENOME ANNOUNCEMENT

Of the 50 currently valid species in the genus *Enterococcus* (1) (http://www.bacterio.cict.fr), many are found in the intestinal tracts of animals (2). The genomic methods used to describe the novel *Enterococcus* species found in the fecal matter of a timber rattlesnake (*E. horridus*, proposed) required sequencing the genomes of closely related enterococci. Here, we describe the sequencing, assembly, and closing of two previously unsequenced *Enterococcus* species genomes, *E. rotai* and *E. silesiacus*.

Genomic DNA of *E. rotai* LMG 26678^T^ and *E. silesiacus* LMG 23085^T^ was isolated and sequenced as previously described (3). Two independent sequencing runs were carried out per isolate. FastQC version 0.10.1 was used to analyze the quality of all the reads. Subsequent work was carried out in CLC Genomics Workbench version 8.5.1. Reads that mapped to the PhiX174 reference or to other organisms sequenced on the same MiSeq run were removed before the reads were trimmed. Any remaining sequencing adapters were removed from the reads. Finally, reads were also trimmed based on ambiguity (ambiguous limit = 2) and quality (limit = 0.02). Assemblies were created and analyzed using the methods of Lauer et al. (3). For both isolates, the assembly produced when K = 55 was selected for further manipulations based on *N*_50_ length and the number of contigs. The *E. silesiacus* assembly had approximately 314× read coverage, whereas the *E. rotai* assembly had an average coverage of 285×. Contigs that had low coverage (≤ 50×) or that were short in length (*n* ≤ 5,000 bp) were discarded. The number of contigs was further reduced through the use of the Genome Finishing Module in CLC Genomics Workbench. Briefly, consensus sequences were extracted from the contigs, and reads were mapped back to these sequences and reassembled before the contigs were extended. After five extensions, the contigs were joined based on sequence overlap. This process was repeated until no more extensions or concatenations were possible. The contigs were then converted into *in* *silico* fragments cut at AflII restriction sites. Single circular optical maps were generated after treating the DNA with AflII and characterizing the fragments using the ARGUS whole-genome optical mapping system (OpGen, Gaithersburg, MD). The *in silico* maps generated from the reads were mapped to the isolate-specific optical map and ordered in MapSolver version 3.2.0. Contigs were placed based on cut patterns, ordered, and extended before being joined manually to produce closed genomes.

### Accession number(s).

These whole-genome shotgun (WGS) projects have been deposited in GenBank under the accession numbers CP013655 (*E. rotai*) and CP013614 (*E. silesiacus*). The assemblies described in this paper are the first versions.

## References

[1] Euzéby JP 1997 List of bacterial names with standing in nomenclature: a folder available on the internet. Int J Syst Bacteriol 47:590–592. doi:10.1099/00207713-47-2-590. 9103655

[2] Devriese LA, Pot B 1995 The genus *Enterococcus*, p 327–367. *In* Wood BJB, Holzapfel WH (ed), The genera of lactic acid bacteria. Blackie Academic & Professional, London, United Kingdom.

[3] Lauer AC, Nicholson AC, Humrighouse BW, Emery B, Drobish A, Juieng P, Loparev V, McQuiston JR 2015 Genome sequences of *Oblitimonas alkaliphila* gen. nov. sp. nov. (proposed), a novel bacterium of the *Pseudomonadaceae* family. Genome Announc 3(6):e01474-15. doi:10.1128/genomeA.01474-15. PMC4683230 26679585

